# Amyloid accumulation, brain atrophy, and cognitive decline in emergent Alzheimer's disease

**DOI:** 10.1002/dad2.70155

**Published:** 2025-07-29

**Authors:** Ying Xia, Pierrick Bourgeat, Vincent Doré, Jurgen Fripp, Yen Ying Lim, Simon M. Laws, Christopher Fowler, Christopher C. Rowe, Colin L. Masters, Elizabeth J. Coulson, Paul Maruff, for the AIBL Research Group

**Affiliations:** ^1^ The Australian e‐Health Research Centre CSIRO Health and Biosecurity Herston Queensland Australia; ^2^ School of Biomedical Sciences The University of Queensland St Lucia Queensland Australia; ^3^ Austin Health Heidelberg Victoria Australia; ^4^ The Australian e‐Health Research Centre CSIRO Health and Biosecurity Parkville Victoria Australia; ^5^ Turner Institute of Brain and Mental Health School of Psychological Sciences Monash University Clayton Victoria Australia; ^6^ Centre for Precision Health Edith Cowan University Joondalup Western Australia Australia; ^7^ Collaborative Genomics and Translation Group School of Medical and Health Sciences Edith Cowan University Joondalup Western Australia Australia; ^8^ Curtin Medical School Curtin University Bentley Western Australia Australia; ^9^ The Florey Institute of Neuroscience and Mental Health The University of Melbourne Parkville Victoria Australia; ^10^ Queensland Brain Institute The University of Queensland St Lucia Queensland Australia; ^11^ Cogstate Ltd. Melbourne Victoria Australia

**Keywords:** Alzheimer's disease, amyloid accumulation, basal forebrain, cognitive decline, longitudinal study, MRI

## Abstract

**INTRODUCTION:**

Emergent Alzheimer's disease (AD) represents a transitional stage where cognitively unimpaired (CU) individuals exhibit subthreshold but increasing amyloid‐β (Aβ) levels. The impact of Aβ accumulation on brain volume loss and cognition during this early stage remains unclear.

**METHODS:**

This retrospective cohort study analyzed data from 408 CU participants who were initially Aβ− (< 15 Centiloids) and followed for up to 15 years. Changes in basal forebrain and hippocampal volume, along with domain‐specific cognitive performance, were compared between those who progressed to Aβ+ (≥20 Centiloids) and those who remained Aβ−.

**RESULTS:**

Sixty‐five CU participants progressed to Aβ+, indicating emergent AD, and showed faster Aβ accumulation and subtle memory decline. However, no significant differences in rate of BF and hippocampal atrophy were observed between groups.

**DISCUSSION:**

The results suggest that during this emergent phase of AD, Aβ accumulation is associated with episodic memory loss, in the absence of detectable accelerated brain atrophy.

**Highlights:**

- Identified cognitively unimpaired individuals in the emergent stage of Alzheimer's disease (AD).
- Emergent AD exhibits a greater rate of amyloid‐β (Aβ) accumulation.
- No accelerated volume loss detected in the basal forebrain or hippocampus.
- Emergent AD is also associated with a subtle decline in memory.
- Early Aβ accumulation may impair cognitive function before structural atrophy.

## Graphical abstract

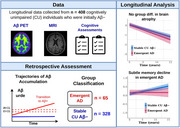

## 1 BACKGROUND

Biological changes that characterize Alzheimer's disease (AD) begin decades before the onset of clinical symptoms, with accumulation of amyloid‐β (Aβ) being a central early change.^1^, ^2^ As a consequence, cognitively unimpaired (CU) older adults with abnormal levels of Aβ (Aβ+), often determined from positron emission tomography (PET) scans with the criterion for abnormality ranging between 15 and 25 Centiloids (CL),^3^, ^4^, ^5^ are classified as having preclinical AD.^6^ This definition of preclinical AD has allowed development of secondary prevention clinical trials to assess whether anti‐amyloid drugs limit further Aβ accumulation.^7^ It also provides a strong biological criterion for understanding AD pathogenesis before Aβ+ can be classified.^8^, ^9^ For example, prospective studies of CU adults with subthreshold Aβ levels (Aβ−) show Aβ accumulation in a small subset of adults,^10^, ^11^ with this more likely to occur in adults who carry at least at least one Apolipoprotein E (*APOE*) ε4 allele.^9^, ^12^ In CU Aβ− adults, Aβ accumulation has been associated only weakly with other AD‐related biological (e.g., tau accumulation, regional brain atrophy) and cognitive changes.^11^, ^13^, ^14^ These weak relationships suggest that Aβ+ is a necessary condition for brain atrophy or cognitive decline, or it might indicate that such changes are truly small and therefore difficult to detect, particularly when there is restriction in the range of subthreshold values for Aβ and other biomarkers,^8^ small sample sizes (*n* < 30),^13^, ^14^ and relatively short study durations (< 4 years).^13^, ^14^

Greater precision for the characterization of the emergence of AD could be achieved by selecting larger numbers of CU adults who meet criteria for preclinical AD and for whom retrospective biomarker and clinical data are available from repeated assessments conducted while they were classified as Aβ−. Rates of change in brain volume and cognition in this emergent AD group could be compared to those of matched CU Aβ− adults whose Aβ status has remained stable over the same time. A starting point in developing a clinical‐pathological model of emergent AD is to measure the extent to which Aβ accumulation across the subthreshold range is associated with brain volume loss and cognitive decline. Magnetic resonance imaging (MRI) studies have identified significant atrophy in the hippocampus and basal forebrain (BF) in preclinical AD.^15^, ^16^, ^17^ Furthermore, rates of volume loss in the cholinergic rich nuclei (i.e., the nucleus basalis of Meynert [NBM]) are associated with decline in memory and attention, beyond that explained by hippocampal atrophy.^18^ Adults with preclinical AD show a large magnitude but reversible decline in spatial memory following a single very low dose (0.2 mg subcutaneously [s.c.]) of the muscarinic antagonist scopolamine, indicating pronounced sensitivity to transient cholinergic disruption.^19^, ^20^ This increased vulnerability suggests that cholinergic dysfunction may emerge early in the disease process. These observations have led to proposals that the loss of cholinergic neurons may precede, and even act to promote accumulation of Aβ.^21^, ^22^, ^23^ This hypothesis could be examined within an emergent AD model by determining the nature of relationships between Aβ accumulation, loss of BF cholinergic neurons, and cognition in CU adults prior to their classification of preclinical AD.

## 2 METHODS

### 2.1 Participants

Participants aged over 60 years without cognitive impairment (*n* = 408) were selected from the Australian Imaging, Biomarker and Lifestyle (AIBL) study of ageing. The AIBL study is a longitudinal observational study designed to improve understanding of AD pathogenesis and diagnosis through psychometric, neuroimaging, and biomarker assessments, with a focus on early detection. Its methodology has been described extensively in the literature, with AIBL participants recruited from two metropolitan study centres in Australia and undergoing comprehensive assessments at 18‐month intervals.^24^, ^25^ Exclusion criteria included a history of non‐AD dementia, Parkinson disease, schizophrenia, bipolar disorder, obstructive sleep apnea, serious head injury, current depression, or a high cardiovascular disease burden (including symptomatic stroke, uncontrolled diabetes, or current regular alcohol consumption exceeding two standard drinks per day for women or four per day for men). The AIBL study was approved by institutional ethics committees of Austin Health, St. Vincent's Health, Hollywood Private Hospital and Edith Cowan University. Written informed consent was obtained from all volunteers before participation and at each visit.

Additional selection criteria included the completion of brain imaging (Aβ‐PET and MRI) and cognitive assessments during the same visit, termed the baseline visit. At baseline, all participants were classified as CU, defined by a Clinical Dementia Rating (CDR) global score of 0, and their Aβ levels were below 15 CL. Additionally, they had attended at least one subsequent visit for Aβ‐PET, MRI, and cognitive assessment. While assessments across modalities were not required to be collected at the same visit, longitudinal data were collected up to the most recent time point at which all three assessments were completed within a 12‐month window. This approach provides sufficient temporal alignment across modalities to support robust longitudinal analyses. *APOE* ε4 status was determined as previously described,^25^ where participants with one or two copies of the *APOE* ε4 allele were classified as *APOE* ε4 carriers and others were classified as non‐carriers.

### 2.2 Neuropsychological assessment

At each assessment visit, participants underwent a comprehensive battery of clinical and neuropsychological tests that covered the main domains of cognition affected by AD and other dementias.^25^ Analyses of cognition focused on episodic memory (hereafter termed *memory*), attention, and executive function, as these domains are known to be impaired in early AD and could be influenced by cholinergic dysfunction.^26^, ^27^, ^28^ Individual cognitive tests included in the neuropsychological battery were first standardized using the mean and standard deviation (SD) scores from a large normative database of CU Aβ− older individuals in AIBL. The domain‐specific composite scores were calculated by averaging sample‐based z‐scores of the relevant cognitive measures as follows: *Memory*—California Verbal Learning Test second edition long delay, logical memory II, and Rey Complex Figure Test 30‐min delay; *Attention*—Digit symbol coding and Digit span tasks (forwards plus backwards); *Executive Function* – Letter fluency test and Category switching total correct.^25^, ^29^

### 2.3 Brain imaging

Aβ‐PET imaging was performed using four different radiotracers: ^18^F‐NAV4694 (43.7%), ^11^C‐Pittsburgh compound‐B (37.4%), ^18^F‐flutemetamol (12.0%), and ^18^F‐florbetapir (6.8%). The imaging methods for Aβ‐PET tracers have been described elsewhere.^18^

**RESEARCH‐IN‐CONTEXT**

1. **Systematic review**: The authors conducted a PubMed search to identify studies examining early changes in brain structure and function in cognitively unimpaired (CU) older adults with subthreshold but increasing amyloid‐β (Aβ) levels, indicating the emergent stage of Alzheimer's disease (AD).
2. **Interpretation**: Our study identifies a relatively large sample of individuals whose AD was emerging. This emergent AD group exhibited faster Aβ accumulation and memory decline but did not show accelerated volume loss in the basal forebrain and hippocampus. These findings suggest that in CU individuals, Aβ accumulation during the emerging phase is associated with memory decline, in the absence of detectable brain atrophy in the basal forebrain and hippocampus.
3. **Future directions**: Future research should validate these findings in larger, more diverse cohorts and investigate mechanisms linking early Aβ accumulation to memory decline through assessment of biomarkers of neuronal and synaptic function or pharmacological challenge studies targeting early AD processes.

MRI scans were acquired using a 3D T1‐weighted magnetization‐prepared rapid gradient‐echo sequence, with the majority using the parameters: repetition time = 2300 *ms*, echo time = 2.98 or 3.05 *ms*, flip angle = 9°, voxel size 1.2 × 1 × 1 *mm^3^* or 1 × 1 × 1 *mm^3^*.

### 2.4 Imaging data processing

Brain Aβ burden was quantified automatically from PET scans using CapAIBL^30^ and estimated in CL values.^31^ Details of PET quantification and harmonization are provided in the eMethods. Aβ status was classified as Aβ+ at levels ≥ 20 CL, and levels < 15 CL were classified as Aβ−. The threshold of ≥ 20 CL aligns with autopsy studies demonstrating that this level corresponds to moderate‐to‐frequent neuritic plaques and reflects a clinically and biologically meaningful Aβ deposition.^32^, ^33^

A longitudinal MRI processing pipeline based on the computational anatomy toolbox (CAT12) was used to process serial MRI scans of each participant.^16^, ^34^ Volumetric measures of the hippocampus and BF subregions were calculated as detailed in eMethods. Analyses of brain volume in the BF focused on two subregions, that is, the posterior subdivision of the NBM (Ch4p) and the medial septal nucleus and vertical limb of the diagonal band of Broca (Ch1/Ch2). These subregions were selected for their distinct patterns of cortical innervations, where Ch4p projects primarily to the temporal cortex and Ch1/Ch2 to the hippocampus, and their different susceptibilities to AD pathology.^16^, ^35^ Studies have demonstrated early Ch4p atrophy in preclinical AD,^17^, ^36^ whereas Ch1/Ch2 shows comparatively less atrophy, serving as a useful reference region to distinguish localized volume loss from global atrophic processes in the BF.^16^

Multi‐scanner effects on brain volumetric measures were corrected using longComBat,^37^ followed by the adjustment for total intracranial volume using regression coefficients estimated from CU Aβ− participants in AIBL. Z‐scores of Ch4p, Ch1/Ch2, and hippocampal volumes were calculated using the mean and SD of the baseline sample, enabling direct comparison of the magnitude of volume change across brain regions.

### 2.5 Statistical analysis

To determine if volume loss in the BF and hippocampus, and cognitive decline occurs in emergent AD, longitudinal data from CU individuals who were Aβ− at baseline were analyzed. CU participants whose Aβ levels increased to meet the criterion for Aβ+ (≥ 20 CL) were classified as *“emergent AD,”* while those whose Aβ levels remained < 15 CL during the study period were classified as *“stable CU Aβ−”*. Individuals in both groups maintained a CDR score of 0 throughout the study period. Participants whose Aβ levels increased above 15 CL but did not reach 20 CL were classified as equivocal and excluded. Baseline group comparisons were performed using one‐way analysis of variance (ANOVA) for continuous data and Pearson's χ^2^ test for categorical data. All analyses were conducted in the R environment (version 4.2.0).

Rates of change in Aβ accumulation, Ch4p, Ch1/Ch2, and hippocampal volume, as well as memory, attention, and executive function were compared between stable CU Aβ− and emergent AD groups using linear mixed‐effects models (LMMs) with the R package *lme4*. Within these models, measures of interest across all repeated visits were submitted as dependent variables, while time from baseline (in years), group status, and their interaction term, group‐by‐time, were entered as fixed factors. Participant and time from baseline were included as random factors. Trajectories of change between the stable CU Aβ− and emergent AD groups were then compared within the LMMs.

To ensure the robustness of the LMM findings, several sensitivity analyses were conducted, as detailed in the eMethods. These included testing alternative Aβ+ cutoffs for defining emergent AD, excluding follow‐up data points with substantial Aβ burden (≥ 40 CL), and evaluating non‐linear model fits. Additionally, exploratory analyses were performed to assess whether trends were more pronounced in the subgroup of *APOE* ε4 carriers.

The relationships between change in brain volume and cognition were further assessed using partial correlation analyses in stable CU Aβ− and emergent AD groups, respectively. To further investigate potential changes that may occur prior to Aβ+, the LMM analyses were repeated in stable CU Aβ− individuals, stratified by *APOE* ε4 carriage status. Given the association between *APOE* ε4 carriage and early Aβ accumulation,^9^, ^12^ this analysis aimed to determine whether CU Aβ− *APOE* ε4 carriers exhibited similar patterns of change in brain volume or cognition compared to non‐carriers.

Age at baseline, sex, and education were included as covariates. Statistical significance was set at *p* < 0.05, with multiple testing corrected using the Benjamini–Hochberg method. Effect sizes (Cohen's *d*) were calculated to quantify group differences: small (0.2–0.5), moderate (0.5–0.8), and large (≥0.8).

## 3 RESULTS

Of the 408 participants (71.6 ± 5.3 years old, 239 [58.6%] female) who satisfied entry criteria, 65 were identified as emergent AD, 15 were excluded from the analysis, and the remaining 328 were classified as stable CU Aβ−. Compared to the stable CU Aβ− group, the emergent AD group had a slightly longer follow‐up, a higher prevalence of *APOE* ε4 carriage and higher education, and higher baseline Aβ burden (Table 1). Groups did not differ in vascular risk factors (e.g., hypertension, diabetes), mood scores, baseline brain volumes or cognitive performance.

**TABLE 1:** Participant characteristics at the baseline visit.

Parameter	Stable CU Aβ− (*n* = 328)	Emergent AD (*n* = 65)	*p*	Cohen's *d / ϕ* ^†^
Age, years, mean (SD)	71.4 (5.4)	72.3 (5.3)	0.226	−0.165
Follow‐up, years, mean (SD)	6.5 (3.6)	8.7 (3.7)	**< 0.001**	**−0.606**
Gender, female, *n* (%)	198 (60.4%)	36 (55.4%)	0.542	0
Education, ≤ 12 years, *n* (%)	112 (34.2%)	34 (52.3%)	**0.009**	**0.130**
Hypertension, *n* (%)	111 (33.8%)	26 (40.0%)	0.418	0
Diabetes, *n* (%)	20 (6.1%)	4 (6.2%)	1.000	0
HADS Depression, mean (SD)	2.23 (1.86)^‡^	1.84 (1.75)^§^	0.144	0.216
HADS Anxiety, mean (SD)	3.88 (2.72)^‡^	3.80 (2.85)^§^	0.843	0.029
*APOE* ε4 carriage, *n* (%)	53 (16.0%)	26 (40.0%)	**< 0.001**	**0.215**
Aβ burden, CL, mean (SD)	−2.3 (5.8)	4.7 (6.7)	**< 0.001**	**−1.176**
Memory, mean (SD)	0.19 (0.67)	0.21 (0.64)	0.856	−0.025
Attention, mean (SD)	0.05 (0.74)	−0.01 (0.73)	0.553	0.081
Executive Function, mean (SD)	0.12 (0.80)	0.09 (0.87)	0.760	0.041
Ch4p, mm^3^, mean (SD)	96.7 (8.2)	96.0 (7.8)	0.575	0.076
Ch1/Ch2, mm^3^, mean (SD)	63.2 (6.4)	63.5 (6.5)	0.680	−0.056
Hippocampus, mm^3^, mean (SD)	6135 (439)	6118 (448)	0.775	0.039

*Note*: Group differences were assessed using one‐way analysis of variance tests for continuous data and χ2 testing for categorized data. Cohen's *d* indicates the magnitude of the group difference. Test statistics are highlighted in bold when significant group differences are observed.

^†^ Effect sizes were assessed using Cohen's d for continuous variables and the phi (ϕ) coefficient for categorical variables.

^‡^ Data on HADS scores were missing for 51 individuals in stable CU Aβ− group.

^§^ Data on HADS scores were missing for 11 individuals in emergent AD group.

Abbreviations: Aβ, amyloid‐β; AD, Alzheimer's disease; *APOE*, Apolipoprotein E; Ch1/Ch2, medial septal nucleus/vertical limb of the diagonal band of Broca; Ch4p, posterior subdivision of nucleus basis of Meynert; CL, Centiloid; CU, cognitively unimpaired; HADS, Hospital Anxiety and Depression Scale; SD, standard deviation.

**FIGURE 1:**
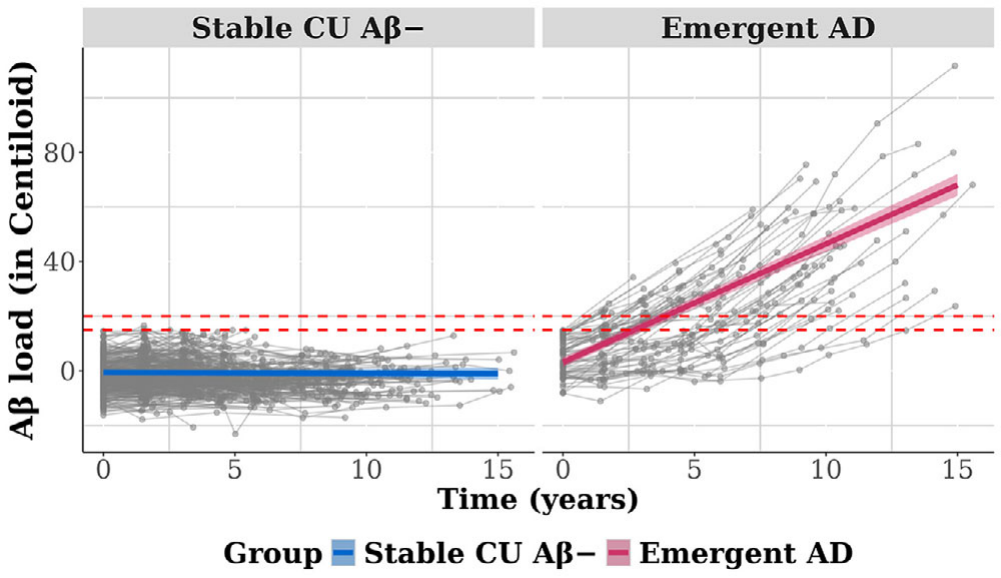
Trajectories of brain Aβ burden for all study participants stratified by group. The red dashed lines indicate the thresholds of Aβ levels at 15 and 20 CL. The gray lines show the individual trajectories of Aβ burden for each participant. The colored lines show the average group trajectories of Aβ burden as estimated using the linear mixed‐effects model. Aβ, amyloid‐β; AD, Alzheimer's disease; CL, Centiloid; CU, cognitively unimpaired.

**FIGURE 2:**
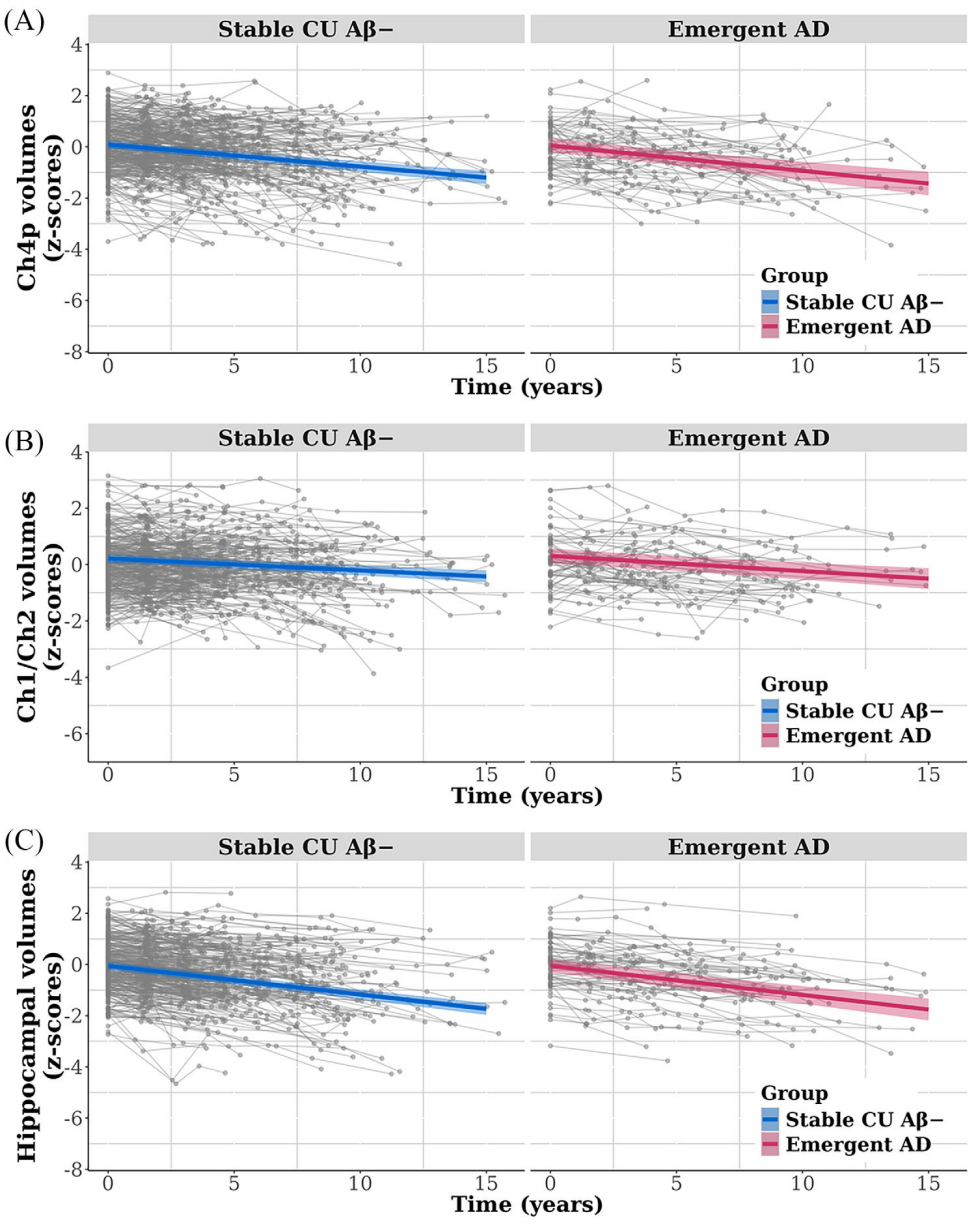
Trajectories of change over time for brain volumetric measures of (A) Ch4p, (B) Ch1/Ch2, and (C) hippocampus, stratified by group. The colored lines show the average group trajectories of changes in brain volumes as estimated using linear mixed‐effects models. The gray lines show the individual trajectories of changes in brain volumes for each participant. Aβ, amyloid‐β; AD, Alzheimer's disease; Ch4p, posterior subdivision of nucleus basalis of Meynert; Ch1/Ch2, medial septal nucleus/vertical limb of the diagonal band of Broca; CU, cognitively unimpaired.

**FIGURE 3:**
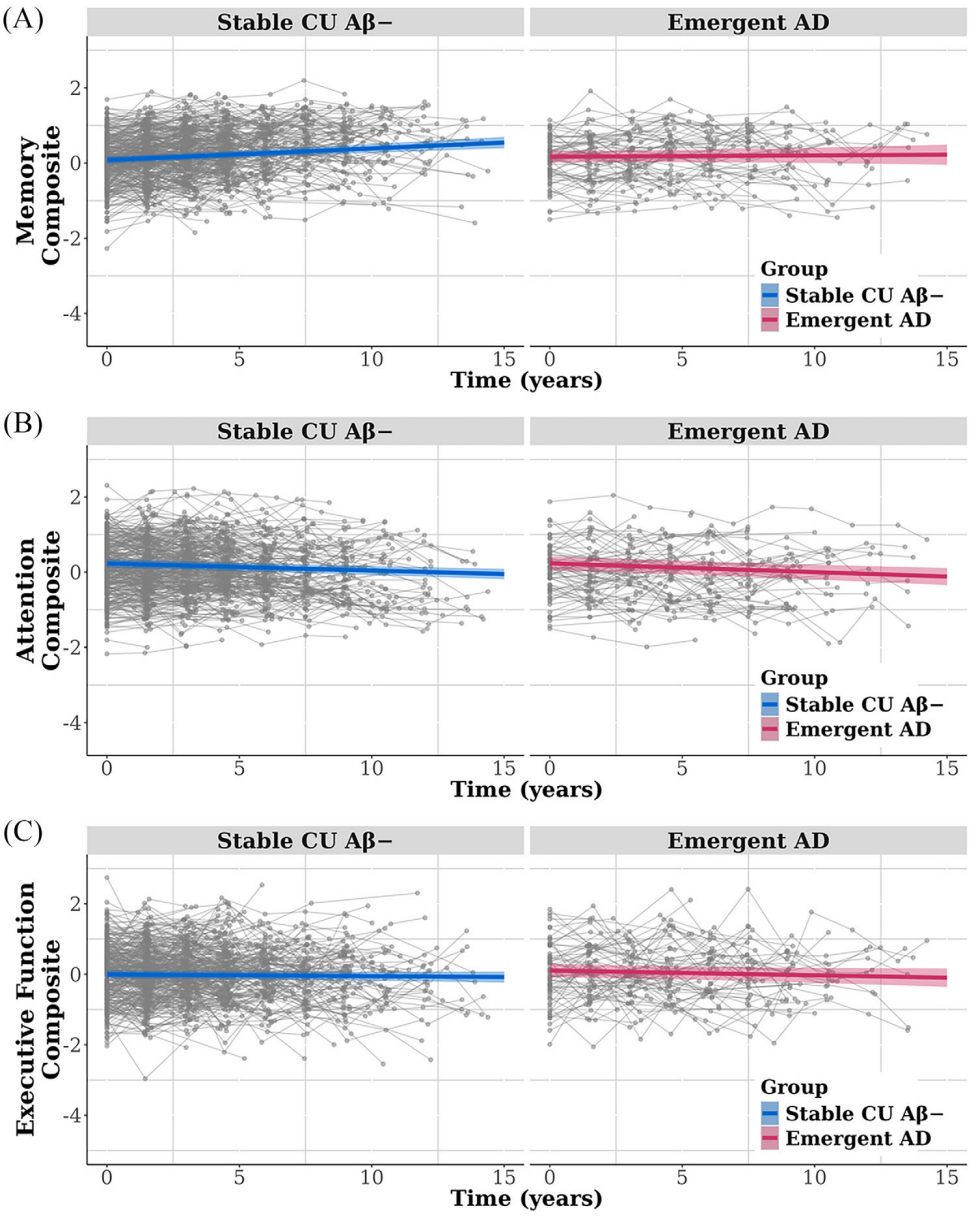
Trajectories of change over time for cognitive scores of (A) memory, (B) attention, and (C) executive function composites, stratified by group. The colored lines show the average group trajectories of changes in cognitive composite scores as estimated using linear mixed‐effects models. The gray lines show the individual trajectories of changes in cognitive composite scores for each participant. Aβ, amyloid‐β; AD, Alzheimer's disease; CU, cognitively unimpaired.

Figure 1 illustrates the trajectories of Aβ accumulation in stable CU Aβ− and emergent AD groups. Table 2 summarizes the results of the LMMs. As expected, the emergent AD group showed a significantly greater rate of Aβ accumulation (mean [SD] change of 4.326 [2.365] CL/year) than the stable CU Aβ− group (−0.032 [0.896] CL/year). No significant differences in rate of volume loss in either BF subregion or the hippocampus was detected between groups (Figure 2). Magnitudes of group differences in rate of volume loss in these brain regions were very small (all *d* ≤ 0.062, Table 2). Comparison of cognitive changes between stable CU Aβ− and emergent AD groups yielded a small but statistically significant difference in memory (Figure 3A), with the emergent AD group showing a subtle decline in performance on the memory composite (annual change of −0.001 [0.070]) and the stable CU Aβ− group showing slight improvement (annual change of 0.031 [0.055]). No differences in rate of change were observed between groups on attention (Figure 3B) or executive function composite scores (Figure 3C).

**TABLE 2:** Linear mixed‐effects models examining rates of change between groups for Aβ burden, volumetric measures of Ch4p, Ch1/Ch2, and hippocampus, as well as composite scores of memory, attention, and executive function.

Parameter	Fixed effect	β (SE)	*p‐Value*	Cohen's *d*
*Group Contrast: emergent AD v.s. stable CU Aβ−*				
Aβ burden	Time	−0.008 (0.018)	0.635	–
	Group × Time	1.149 (0.039)	**< 0.001**	**−1.740**
Ch4p volume	Time	−0.296 (0.026)	**< 0.001**	–
	Group × Time	−0.044 (0.056)	0.429	0.057
Ch1/Ch2 volume	Time	−0.145 (0.021)	**< 0.001**	–
	Group × Time	−0.040 (0.045)	0.378	0.062
Hippocampal volume	Time	−0.386 (0.021)	**< 0.001**	–
	Group × Time	−0.006 (0.045)	0.895	0.009
Memory composite	Time	0.104 (0.015)	**< 0.001**	–
	Group × Time	−0.092 (0.033)	**0.006**	**0.189**
Attention composite	Time	−0.062 (0.012)	**< 0.001**	–
	Group × Time	−0.016 (0.024)	0.514	0.049
Executive function composite	Time	−0.017 (0.015)	0.281	–
	Group × Time	−0.029 (0.031)	0.359	0.076

*Note*: Group comparisons were made with the latter group serving as the reference. β represents the standardized regression coefficients. All *p* values are reported before correction for multiple testing. Statistical outputs are highlighted in bold if they remain statistically significant after correcting for multiple testing.

Abbreviations: Aβ, amyloid‐β; AD, Alzheimer's disease; Ch1/Ch2, medial septal nucleus/vertical limb of the diagonal band of Broca; Ch4p, posterior subdivision of nucleus basalis of Meynert; CU, cognitively unimpaired; SE, standard error.

The results were consistent when emergent AD was defined using alternative Aβ+ cutoffs of 15 and 25 CL (eTable 1), as well as in sensitivity analyses restricted to assessments with Aβ levels < 40 CL (eFigure 1). Although non‐linear models better captured longitudinal trajectories of brain measures except executive function composite scores (eTable 2), significant group effects were observed in the slope and curvature of Aβ accumulation trajectories, as well as in the slope of the memory decline (eTable 3 and eFigures 2‐4). Exploratory analyses revealed similar longitudinal patterns across all brain measures within *APOE* ε4 carriers (eTable 4), with a notably greater effect size observed for group differences in memory trajectories.

**FIGURE 4:**
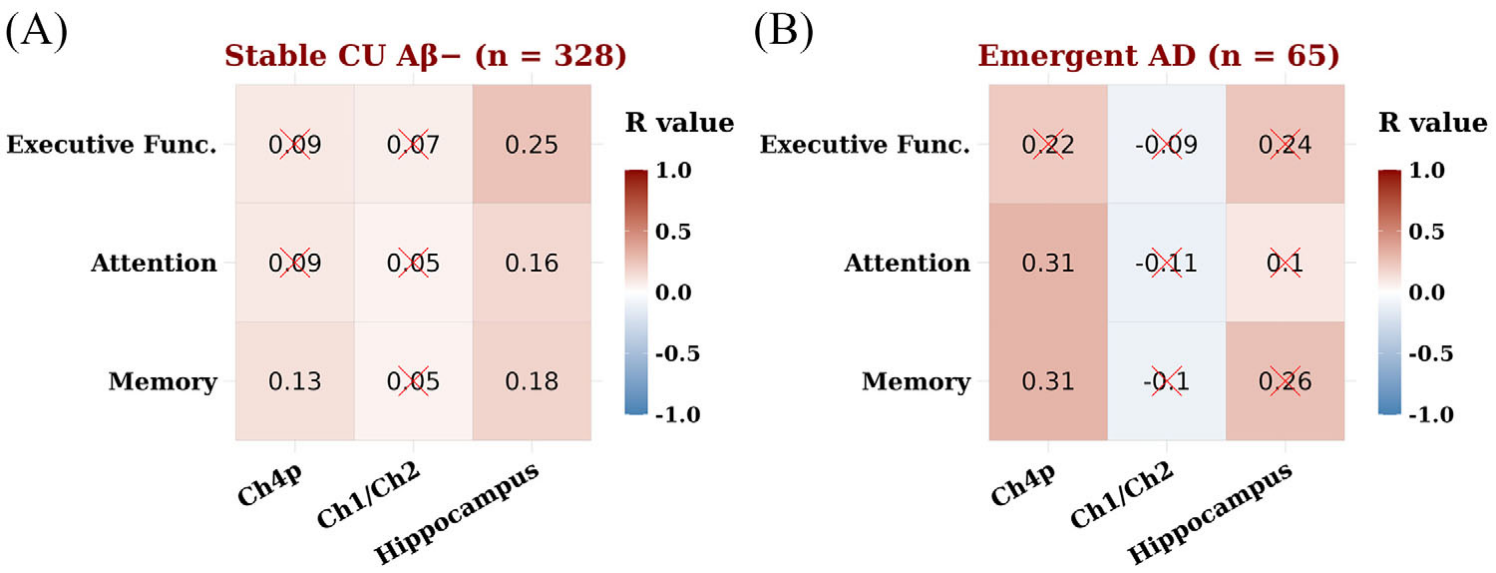
Partial correlation analysis reveals associations between regional brain atrophy and cognitive decline in (A) stable CU Aβ− individuals and (B) those with emergent AD. Each heatmap illustrates Pearson correlation coefficients (*R* values), indicating the effect size and direction of associations between rates of volumetric change in Ch4p, Ch1/Ch2, and hippocampus and rates of change in composite scores of memory, attention, and executive function. All correlation analyses were adjusted for age, sex, and education. Correlations that do not achieve statistical significance (*p* < 0.05, adjusted for multiple testing) are denoted by a red cross. Aβ, amyloid‐β; AD, Alzheimer's disease; Ch4p, posterior subdivision of nucleus basalis of Meynert; Ch1/Ch2, medial septal nucleus/vertical limb of the diagonal band of Broca; CU, cognitively unimpaired.

The results of the partial correlation analysis are summarized in Figure 4 and eTable 5 and that in the stable CU Aβ− group, Ch4p volume loss was associated weakly but significantly with change in memory (*r* = 0.13, *p* = 0.016). Hippocampal volume loss was associated moderately and significantly with change across three cognitive domains. In the emergent AD group, Ch4p volume loss correlated more strongly with change in memory (*r* = 0.31, *p* = 0.013; eFigure 5) and attention (*r* = 0.31, *p* = 0.014). Volume loss in the hippocampus or Ch1/Ch2 was not associated with changes in cognition.

Repeating the LMM analyses to compare changes in Aβ burden, brain volume, and cognition between CU Aβ− *APOE* ε4 carriers (*n* = 53) and non‐carriers (*n* = 275), identified that *APOE* ε4 carriers exhibited a significant but small magnitude increase in the rate of Aβ accumulation compared to non‐carriers (eTable 6), although by definition no adult in this group had Aβ levels reach the criterion for Aβ+ during the study period (eFigure 6). No significant group differences were observed for rates of change in brain volume or cognition (eTable 6).

## 4 DISCUSSION

Analysis of biomarker, MRI, and cognitive data from CU adults enrolled in the AIBL study, who currently meet criteria for preclinical AD (i.e., CU with Aβ levels ≥ 20 CL) and for whom retrospective PET assessments of Aβ levels showed these to be within normal limits (< 15 CL), allowed identification of a relatively large sample of older individuals (*n* = 65) whose AD was emerging (Figure 1). The emergent AD group was aged approximately 72 years old at baseline and provided relevant data over an average 8‐year follow‐up period. This approach to classification also provided a large group of individuals who remained CU Aβ− throughout the study period. Although baseline Aβ levels were ∼7 CL higher in the emergent AD group than in the stable CU Aβ− group, Aβ levels in both groups were close to zero with substantial overlap between their estimates of variance (Table 1). Consistent with the known higher risk of Aβ accumulation associated with *APOE* ε4 carriage,^9^, ^12^ the emergent AD group contained more ε4 carriers.

As expected, the emergent AD group showed much faster Aβ accumulation than the stable CU Aβ− group, providing a marked difference in accumulation trajectories (Figure 1). This large difference provided a strong framework for understanding how amyloid accumulation affects brain volume and cognition over the same time interval, particularly when compared to previous studies with smaller CU Aβ− samples classified as Aβ accumulators based on shorter intervals and fewer observations.^13^, ^14^ Within this framework, subtle memory decline was identified in the emergent AD group, while rates of volume loss in the hippocampus and BF subregions, as well as changes in attention and executive function, did not differ between groups (Table 2). Existing studies show that CU Aβ+ individuals (i.e., preclinical AD) experience subtle but relentless cognitive decline, primarily in memory,^29^, ^38^, ^39^, ^40^ along with progressive atrophy in the hippocampus and BF, particularly the Ch4p subregion.^16^, ^17^ The current data indicate that, while substantial amyloid accumulation begins many years before the classification of Aβ+, the downstream effects of this are minimal in terms of brain volume loss, at least in the hippocampus and BF, as well as cognition.

The finding that, despite faster Aβ accumulation, emergent AD was not associated with increased volume loss in BF cholinergic‐rich areas or the hippocampus (Figure 2), indicates that early Aβ accumulation precedes the development of atrophy in these regions, at least to the extent detected by MRI. However, emergent AD was associated with a subtle loss of memory, despite no detectable accelerated atrophy in the BF and hippocampus, two regions associated strongly with cognitive decline in AD.^18^, ^29^ This dissociation suggests that increasing Aβ burden may disrupt neuronal function before structural degeneration becomes apparent. Subtle Aβ‐related memory dysfunction, detected through repeated neuropsychological assessments in Aβ− individuals, has been observed previously.^10^, ^14^ However, these studies were limited by short follow‐up periods, small sample sizes,^14^ or reliance on mathematical models of Aβ accumulation^10^ rather than from actual data. Memory decline without accelerated brain atrophy may reflect a direct toxic effect of amyloid on neurons or Aβ‐related disruption to neuronal function, including cholinergic neurotransmission, prior to measurable neuronal loss.^41^ These hypotheses can be challenged in emergent AD using biomarkers of neuronal and synaptic function or through active pharmacological challenge studies such as those previously applied in preclinical AD.^19^

The small difference in memory decline between groups occurred because the stable CU Aβ− group showed a subtle improvement in performance in their memory composite scores, while the emergent AD group did not. An absence of improvement from repeated application of neuropsychological tests, termed practice effects, is a common characteristic of longitudinal samples of preclinical AD.^42^, ^43^ Reduced practice effects in preclinical AD have been reconceptualized as an AD‐related learning impairment.^42^ Guided by this theoretical perspective, a 6‐day learning paradigm revealed impairments in preclinical AD, at a magnitude four times greater than longitudinal estimates from 10‐year repeated neuropsychological assessments.^42^ Similar learning deficits were observed in CU Aβ− *APOE* ε4 carriers compared to matched non‐carriers.^44^ These observations converge to support the hypothesis that in CU adults, Aβ accumulation during the emerging phase is associated with impaired learning, even in the absence of any detectable accelerated loss of the BF or hippocampal volume.

This study leveraged a unique group of CU individuals with emergent AD, as defined by a clear transition to Aβ+, to assess the potential brain volume and cognitive changes associated with initial Aβ accumulation. While this work relied on the retrospective classification of emergent AD, limiting its immediate applicability for prospective identification of individuals at risk, it serves as a proof‐of‐concept for tracking neurobiological changes during this very early disease stage. Recognizing that Aβ accumulation is a gradual process and that pathophysiological effects may begin before thresholds for abnormality are reached, this approach supports the development of more sophisticated, prospective strategies for early detection. The emergent AD group identified here could serve as a promising target for future work seeking to refine predictive models and evaluate early interventions and prevention strategies for AD.

Some caveats limit the generalizability of the study. MRI‐derived volumetric measures capture gross tissue changes related to BF cholinergic neuronal loss but are not sensitive to subtle neuronal or synaptic dysfunction. These measures may also exhibit greater variability, particularly in subregions, due to methodological challenges, which may limit their sensitivity to early and subtle neurodegenerative changes. Application of advanced cholinergic imaging could provide insight into early Aβ‐related cholinergic alterations in emergent AD.^41^, ^45^ Second, while participants presented initially as CU Aβ−, it remains uncertain whether other AD processes, for example, tau pathology and neuroinflammation, were also present during this period and may have contributed to early cognitive changes. Future studies with more comprehensive data will be essential to better characterize this disease stage. Third, using ≥ 20 CL for Aβ+ may not fully capture the subtle dynamics of Aβ accumulation in the emergent phase. However, the clinical‐pathological framework for emergent AD developed here provides a strong basis for challenging biological processes associated with amyloid accumulation before the preclinical AD stage, their clinical manifestations, and the extent to which such processes could be modified by anti‐amyloid therapies. Lastly, it is important to emphasize that the AIBL study was designed to investigate how amyloid accumulation influences the clinical expression of AD. Accordingly, exclusion criteria for the study are directed at minimizing the effects of other age‐related brain changes that might also influence cognitive and behavioral symptoms including cardiovascular and cerebrovascular diseases. Consequently, AIBL participants are a highly motivated, well‐educated group of volunteers, with a low prevalence of health comorbidities.^25^ While this aspect of the AIBL sample facilitates understanding of the emergence of AD, it does limit the generalizability of these findings and makes replication necessary in other natural history studies.

In conclusion, findings of this retrospective study suggest that, during the emergent stage of AD, Aβ accumulation occurs in the absence of measurable accelerated atrophy in the BF or hippocampus. The observed memory decline in this emergent phase indicates that Aβ‐related neuronal dysfunction occurs prior to the detectable structural degeneration. Future studies should employ advanced imaging to explore mechanisms linking Aβ pathology to early memory changes in emergent AD.

## CONFLICT OF INTEREST STATEMENT

All authors report no conflicts of interest relevant to this article. Author disclosures are available in the Supporting Information.

## CONSENT STATEMENT

The AIBL study was approved by institutional ethics committees of Austin Health, St. Vincent's Health, Hollywood Private Hospital, and Edith Cowan University. Written informed consent was obtained from all volunteers before participation and at each visit.

## Supporting information



Supporting Information

Supporting Information
