# Supplementary material for: Amyloid accumulation, brain atrophy, and cognitive decline in emergent Alzheimer's disease
Source: Alzheimers Dement (Amst). 2025 Jul 29;17(3):e70155. doi: 10.1002/dad2.70155 (PMC12305117; doi:10.1002/dad2.70155)
Supplement: Supplementary file 1 — Supporting Information [file DAD2-17-e70155-s002.docx]

Supplemental Online Content

**Amyloid accumulation, brain atrophy, and cognitive decline in emergent Alzheimer’s disease**

Ying Xia^*^, Pierrick Bourgeat, Vincent Doré, Jurgen Fripp, Yen Ying Lim, Simon M. Laws, Christopher Fowler, Christopher C. Rowe, Colin L. Masters, Elizabeth J. Coulson, Paul Maruff, for the AIBL Research Group

^*^Correspondence to: [ying.xia@csiro.au](mailto:ying.xia@csiro.au)

**This supplementary document includes:**

- **eMethods**
- **References**
- **eTable 1.** Sensitivity analysis of linear mixed-effects model results based on different Centiloid thresholds for defining the emergent AD group.
- **eTable 2.** Model comparison results for longitudinal trajectories across different brain measures.
- **eTable 3.** Optimal models examining group effects on the intercept, slope, and quadratic curvature of longitudinal trajectories across brain measures.
- **eTable 4.** Linear mixed-effects models repeated within *APOE* ε4 carriers.
- **eTable 5.** Partial correlation analysis of rates of volume loss in brain regions (Ch4p, Ch1/Ch2, and hippocampus) and rates of change in memory, attention, and executive function composite scores across stable CU Aβ− and emergent AD groups.
- **eTable 6.** Linear mixed-effects models examining rates of change between stable CU Aβ− *APOE* ε4 carriers and non-carriers.
- **eFigure 1.** Sensitivity analysis assessing the average group trajectories of brain Aβ burden and memory composite scores in the stable CU Aβ− and emergent AD groups, with data restricted to assessments associated with visits where Aβ levels were below 40 CL.
- **eFigure 2.** Non-linear modelling to longitudinal trajectories of brain Aβ burden for all study participants stratified by group.
- **eFigure 3.** Non-linear modelling of longitudinal trajectories for Ch4p, Ch1/Ch2, and hippocampal volumes, stratified by group.
- **eFigure 4.** Non-linear modelling of longitudinal trajectories for Memory and Attention composite scores, stratified by group.
- **eFigure 5.** Scatterplots showing the associations between the rate of volumetric change in different brain regions and the rate of change in memory.
- **eFigure 6.** Trajectories of brain Aβ burden and memory composite scores for stable CU Aβ− individuals, stratified by *APOE* ε4 carriage.

**eMethods**

*Amyloid-β PET quantification*

The CapAIBL (Computational Analysis of PET by AIBL) method was used to automatically quantify the amyloid-β (Aβ) burden in the brain from positron emission tomography (PET) images. Without requiring magnetic resonance imaging (MRI) scans, PET images were spatially normalized directly to a standard template using an adaptive atlas approach.^1^ The standardized uptake value ratio normalization was performed in standard space using the Centiloid Whole Cerebellum mask.^2^ The non-negative matrix factorization-based quantification method was applied to quantify the Aβ burden in Centiloid (CL) values, enhancing the robustness of longitudinal quantification for Aβ PET images across different tracers in the AIBL study.^3^

*Longitudinal structural MRI processing*

Using the longitudinal MRI processing pipeline in the computational anatomy toolbox (CAT12),^4^ serial MRI scans of the same participant were processed for brain tissue segmentation and spatial normalization to a standard space using an image analysis workflow in CAT12 that incorporated special considerations for age-related changes. Hippocampal volume was calculated by combining the volumes of the bilateral hippocampal regions, identified in standard space using the Neuromorphometrics atlas (<https://www.neuromorphometrics.com>). To identify the basal forebrain (BF) regions, gray matter masks segmented from serial MRI scans were initially aligned to a pre-generated population template using the DARTEL toolbox in a longitudinal registration method.^5^ Subregional BF volumes were calculated in this template space using a stereotactic mask of the bilateral BF,^6^ which included *Ch4p* - the posterior subdivision of the nucleus basalis of Meynert (NBM), *Ch4a_i* – the anterior and intermediate subdivisions of the NBM, *Ch3* – the horizontal limb of the diagonal band of Broca, *Ch1/Ch2* – the medial septal nucleus and vertical limb of the diagonal band of Broca, and the nucleus subputaminalis.

*Sensitivity analyses*

Alternative thresholds for Aβ positivity To assess the potential influence of the CL threshold for Aβ positivity (Aβ+) on the longitudinal analysis results, group classification for emergent Alzheimer’s disease (AD) was redefined using two alternative thresholds, i.e., 15 CL (lower than the original 20 CL threshold reported in the main analysis) and 25 CL (higher than the original threshold). The threshold for Aβ− remained at < 15 CL. Linear mixed-effects (LMM) model analyses were then repeated using these alternative classifications to evaluate the robustness of group-level differences under varying classification criteria for Aβ+.

Exclusion of follow-up data points with substantial Aβ burden For any significant findings reported in the main analysis, a sensitivity analysis was performed excluding follow-up assessments where Aβ levels were ≥ 40 CL, to ensure that results were not disproportionately driven by data points reflecting substantial Aβ burden. This exclusion also helps to minimize the potential influence of tau pathology, as a low prevalence (~10%) of abnormal tau burden in the temporoparietal neocortex was reported among CU individuals with Aβ levels < 40 CL.^7^

Non-linear model fitting To evaluate whether non-linear model fitting better captured longitudinal trajectories of Aβ, brain volumes, and cognitive composite scores, we tested two alternative model specifications (M2 and M3) in comparison to the original linear model (M1), as listed below:

- **M1 (linear)**: *Y ~ Age_BL_ + Sex + Education + Group + Time + Group × Time + (1 + Time | Subject)*
- **M2 (non-linear)**: *Y ~ Age_BL_ + Sex + Education + Group + Time + Time^2^ + Group × Time + (1 + Time | Subject)*
- **M3 (non-linear)**: *Y ~ Age_BL_ + Sex + Education + Group + Time + Time^2^ + Group × Time + Group × Time^2^ + (1 + Time + Time^2^ | Subject)*

*Y* is the dependent variable, which could be the Aβ burden, brain regional volume, or domain-specific composite score*. Time* is the time (in years) since baseline, *Age_BL_* is age at baseline, and *Group* represents the group status, classified as either emergent AD or stable cognitively unimpaired (CU) Aβ−.

The M2 extended the original linear model by including a quadratic term for *Time* (i.e., *Time^2^*). The M3 further extended M2 by including a fixed *Group × Time^2^* interaction to capture potential differences in curvatures between groups and updating the random effects to *(1 + Time + Time^2^ | Subject)* to account for individual variability in intercepts, slopes, and curvatures.

ANOVA tests were conducted to compare nested mixed-effect models and evaluate whether the non-linear models with increased complexity significantly improve fit. Model fit was evaluated using Akaike Information Criterion (AIC), Bayesian Information Criterion (BIC), and Log-Likelihood (LogLik) values. Fixed group effects on the intercept, slope, and curvature of longitudinal trajectories were assessed in the best-fitting models for Aβ accumulation, Ch4p, Ch1/Ch2, and hippocampal volumes, and cognitive composite scores of memory, attention, and executive function.

**References**

1. Bourgeat P, Villemagne VL, Dore V, et al. Comparison of MR-less PiB SUVR quantification methods. *Neurobiol Aging.* 2015;36:S159-S166. doi: 10.1016/j.neurobiolaging.2014.04.033.

2. Klunk WE, Koeppe RA, Price JC, et al. The Centiloid Project: standardizing quantitative amyloid plaque estimation by PET. *Alzheimers Dement.* 2015;11(1):1-15. doi: 10.1016/j.jalz.2014.07.003.

3. Bourgeat P, Doré V, Doecke J, et al. Non-negative matrix factorisation improves Centiloid robustness in longitudinal studies. *NeuroImage.* 2021;226:117593. doi: 10.1016/j.neuroimage.2020.117593.

4. Gaser C, Dahnke R, Thompson PM, Kurth F, Luders E, The Alzheimer's Disease Neuroimaging I. CAT: a computational anatomy toolbox for the analysis of structural MRI data. *Gigascience.* 2024;13:giae049. doi: 10.1093/gigascience/giae049.

5. Ashburner J. A fast diffeomorphic image registration algorithm. *NeuroImage.* 2007;38(1):95-113. doi: 10.1016/j.neuroimage.2007.07.007.

6. Kilimann I, Grothe M, Heinsen H, et al. Subregional Basal Forebrain Atrophy in Alzheimer's Disease: A Multicenter Study. *J Alzheimers Dis.* 2014;40:687-700. doi: 10.3233/JAD-132345.

7. Doré V, Krishnadas N, Bourgeat P, et al. Relationship between amyloid and tau levels and its impact on tau spreading. *Eur J Nucl Med Mol Imaging.* 2021;48(7):2225-2232. doi: 10.1007/s00259-021-05191-9.

| **eTable 1.** Sensitivity analysis of linear mixed-effects model results based on different Centiloid thresholds for defining the emergent AD group. | | | | |
| --- | --- | --- | --- | --- |
| **Aβ+ threshold** | | **≥ 15 CL** | **≥ 20 CL^†^** | **≥ 25 CL** |
| **No. of emergent AD** | | 77 | 65 | 52 |
| **No. of stable CU Aβ−** | | 328 | 328 | 328 |
| **Aβ burden** | **β (SE)** | **1.054 (0.040)^***^** | **1.149 (0.039)^***^** | **1.218 (0.039)^***^** |
|  | ***d*** | **−1.458** | **−1.740** | **−1.979** |
| **Ch4p volume** | **β (SE)** | −0.016 (0.053) | −0.044 (0.056) | −0.014 (0.061) |
|  | ***d*** | 0.022 | 0.057 | 0.017 |
| **Ch1/Ch2 volume** | **β (SE)** | −0.031 (0.043) | −0.040 (0.045) | −0.039 (0.048) |
|  | ***d*** | 0.050 | 0.062 | 0.060 |
| **Hippocampal volume** | **β (SE)** | 0.016 (0.043) | −0.006 (0.045) | −0.005 (0.049) |
|  | ***d*** | −0.024 | 0.009 | 0.007 |
| **Memory composite** | **β (SE)** | **−0.080 (0.031)^*^** | **−0.092 (0.033)^**^** | **−0.078 (0.036)^*^** |
|  | ***d*** | **0.171** | **0.189** | **0.150** |
| **Attention composite** | **β (SE)** | −0.014 (0.022) | −0.016 (0.024) | −0.012 (0.026) |
|  | ***d*** | 0.046 | 0.049 | 0.035 |
| **Executive Function composite** | **β (SE)** | −0.031 (0.029) | −0.029 (0.031) | −0.021 (0.033) |
|  | ***d*** | 0.086 | 0.076 | 0.053 |
| β represents the standardized regression coefficient for the fixed group × time interaction term in the linear mixed effect model. Significant levels for this term are annotated as follows: * *p* < 0.05, ** *p* < 0.001, and *** *p* < 0.001. Cohen’s *d* is reported to indicate effect size. No correction for multiple comparisons was applied, as these analyses were exploratory in nature.  **^†^** Linear mixed effects model results for the threshold of ≥ 20 CL have been reported in Table 2 of the main text.  Abbreviations: Aβ, amyloid-β; Aβ+, abnormal Aβ levels; AD, Alzheimer’s disease; Ch4p, posterior subdivision of nucleus basalis of Meynert; Ch1/Ch2, medial septal nucleus/vertical limb of the diagonal band of Broca; CL, Centiloid; CU, cognitively unimpaired; SE, standard error. | | | | |

| **eTable 2.** Model comparison results for longitudinal trajectories across different brain measures. | | | | | | | |
| --- | --- | --- | --- | --- | --- | --- | --- |
| **Dependent Variable** | **Model** | **Fixed Time Effects** | **AIC** | **BIC** | **LogLik** | **M1 vs. M2** | **M2 vs. M3** |
| **Aβ burden** | M1 | Linear | 9697.3 | 9756.2 | − 4837.7 |  |  |
|  | M2 | Linear + Quadratic | 9579.2 | 9643.5 | − 4777.6 | χ^2^(1) = 120.1  *p* < 0.001 |  |
|  | **M3** | **Linear + Quadratic + Group diff. in curvatures** | **9312.9** | **9398.5** | **−** **4640.4** |  | **χ^2^(4) = 274.3**  ***p* < 0.001** |
| **Ch4p volume** | M1 | Linear | 3090.4 | 3148.4 | − 1534.2 |  |  |
|  | M2 | Linear + Quadratic | 3086.8 | 3150.0 | − 1531.4 | χ^2^(1) = 5.6  *p* = 0.018 |  |
|  | **M3** | **Linear + Quadratic + Group diff. in curvatures** | **3068.4** | **3152.7** | **−** **1518.2** |  | **χ^2^(4) = 26.4**  ***p* < 0.001** |
| **Ch1/Ch2 volume** | M1 | Linear | 2779.7 | 2837.6 | − 1378.8 |  |  |
|  | M2 | Linear + Quadratic | 2764.1 | 2827.3 | − 1370.1 | χ^2^(1) = 17.6  *p* < 0.001 |  |
|  | **M3** | **Linear + Quadratic + Group diff. in curvatures** | **2761.9** | **2846.2** | **−** **1365.0** |  | **χ^2^(4) = 10.2**  ***p* = 0.037** |
| **Hippocampal volume** | M1 | Linear | 2330.1 | 2388.0 | − 1154.0 |  |  |
|  | M2 | Linear + Quadratic | 2330.2 | 2393.5 | − 1153.1 | χ^2^(1) = 1.8  *p* = 0.176 |  |
|  | **M3** | **Linear + Quadratic + Group diff. in curvatures** | **2324.2** | **2408.5** | **−** **1146.1** |  | **χ^2^(4) = 14.0**  ***p* = 0.007** |
| **Memory composite** | M1 | Linear | 2354.3 | 2415.3 | − 1166.2 |  |  |
|  | **M2** | **Linear + Quadratic** | **2332.7** | **2399.2** | **−** **1154.3** | **χ^2^(1) = 23.7**  ***p* < 0.001** |  |
|  | M3 | Linear + Quadratic + Group diff. in curvatures | 2331.7 | 2420.4 | − 1149.9 |  | χ^2^(4) = 8.9  *p* = 0.063 |
| **Attention composite** | M1 | Linear | 2217.1 | 2278.1 | − 1097.6 |  |  |
|  | **M2** | **Linear + Quadratic** | **2210.8** | **2277.3** | **−** **1093.4** | **χ^2^(1) = 8.4**  ***p* = 0.004** |  |
|  | M3 | Linear + Quadratic + Group diff. in curvatures | 2216.2 | 2304.9 | − 1092.1 |  | χ^2^(4) = 2.6  *p* = 0.623 |
| **Executive Func. composite** | **M1** | **Linear** | **3285.4** | **3346.4** | **−** **1631.7** |  |  |
|  | M2 | Linear + Quadratic | 3286.9 | 3353.5 | − 1631.5 | χ^2^(1) = 0.5  *p* = 0.495 |  |
|  | M3 | Linear + Quadratic + Group diff. in curvatures | 3291.9 | 3380.6 | − 1629.9 |  | χ^2^(4) = 3.1  *p* = 0.548 |
| Three nested linear mixed-effects models (M1, M2, M3) were fit to each dependent variable:  *M1: Y ~ Age + Sex + Education + Group + Time + Group × Time + (1 + Time \| Subject).*  *M2: Y ~ Age + Sex + Education + Group + Time + Time² + Group × Time + (1 + Time \| Subject).*  *M3: Y ~ Age + Sex + Education + Group + Time + Time² + Group × Time + Group × Time² + (1 + Time + Time² \| Subject).*  Model fit was assessed using AIC, BIC, and LogLik. Chi-squared (χ²) statistics and *p*-values are from likelihood ratio tests comparing successive models (M1 vs. M2, M2 vs. M3).  Abbreviations: Aβ, amyloid-β; AIC, Akaike Information Criterion; BIC, Bayesian Information Criterion; LogLik, log-likelihood; Ch4p, posterior subdivision of nucleus basalis of Meynert; Ch1/Ch2, medial septal nucleus/vertical limb of the diagonal band of Broca. | | | | | | | |

| **eTable 3.** Optimal models examining group effects on the intercept, slope, and quadratic curvature of longitudinal trajectories across brain measures. | | | | | |
| --- | --- | --- | --- | --- | --- |
| **Dependent Variable** | **Optimal Model** | **Fixed Effect** | **Standardized β** | **Standard Error** | ***p*-value** |
| **Aβ burden** | M3 | Group | 1.330 | 0.074 | **< 0.001** |
|  |  | Group *×* Time | 1.019 | 0.044 | **< 0.001** |
|  |  | Group *×* Time^2^ | 0.242 | 0.018 | **< 0.001** |
| **Ch4p volume** | M3 | Group | −0.073 | 0.139 | 0.602 |
|  |  | Group *×* Time | −0.042 | 0.062 | 0.497 |
|  |  | Group *×* Time^2^ | −0.002 | 0.038 | 0.956 |
| **Ch1/Ch2 volume** | M3 | Group | 0.038 | 0.125 | 0.759 |
|  |  | Group *×* Time | −0.034 | 0.052 | 0.517 |
|  |  | Group *×* Time^2^ | 0.028 | 0.032 | 0.379 |
| **Hippocampal volume** | M3 | Group | 0.015 | 0.136 | 0.911 |
|  |  | Group *×* Time | 0.007 | 0.046 | 0.873 |
|  |  | Group *×* Time^2^ | −0.014 | 0.026 | 0.592 |
| **Memory composite** | M2 | Group | −0.015 | 0.077 | 0.848 |
|  |  | Group *×* Time | −0.077 | 0.033 | **0.021** |
| **Attention composite** | M2 | Group | −0.013 | 0.087 | 0.879 |
|  |  | Group *×* Time | −0.009 | 0.024 | 0.712 |
| **Executive Function composite** | M1 | Group | 0.077 | 0.089 | 0.385 |
|  |  | Group *×* Time | −0.029 | 0.031 | 0.359 |
| Three nested linear mixed-effects models (M1, M2, M3) were fit and compared for each brain measure. The optimal model was selected based on model comparison results for each brain measure. The fixed effects of *Group, Group × Time, and Group × Time^2^* indicate group differences in the intercept, slope, and quadratic curvature of the longitudinal trajectories for each brain measure.  *M1: Y ~ Age + Sex + Education + Group + Time + Group × Time + (1 + Time \| Subject).*  *M2: Y ~ Age + Sex + Education + Group + Time + Time² + Group × Time + (1 + Time \| Subject).*  *M3: Y ~ Age + Sex + Education + Group + Time + Time² + Group × Time + Group × Time² + (1 + Time + Time² \| Subject).*  Abbreviations: Aβ, amyloid-β; Ch4p, posterior subdivision of nucleus basalis of Meynert; Ch1/Ch2, medial septal nucleus/vertical limb of the diagonal band of Broca | | | | | |

| **eTable 4.** Linear mixed-effects models repeated within *APOE* ε4 carriers. In the subgroup of n = 79 *APOE* ε4 carriers, 26 were classified as emergent AD and the remaining 53 remained stable CU Aβ−. | | | | |
| --- | --- | --- | --- | --- |
|  | **Fixed Effect** | **β (SE)** | ***P-value*** | **Cohen’s *d*** |
| *Group Contrast: emergent AD v.s. stable CU Aβ−* | | | | |
| **Aβ burden** | Time | 0.100 (0.056) | 0.079 | - |
|  | Group × Time | 1.105 (0.088) | < 0.001 | −1.503 |
| **Ch4p volume** | Time | −0.311 (0.065) | < 0.001 | - |
|  | Group × Time | −0.091 (0.098) | 0.359 | 0.153 |
| **Ch1/Ch2 volume** | Time | −0.163 (0.054) | 0.004 | - |
|  | Group × Time | −0.033 (0.084) | 0.698 | 0.058 |
| **Hippocampal volume** | Time | −0.429 (0.048) | < 0.001 | - |
|  | Group × Time | −0.019 (0.075) | 0.796 | 0.041 |
| **Memory composite** | Time | 0.110 (0.044) | 0.014 | - |
|  | Group × Time | −0.178 (0.065) | 0.009 | 0.399 |
| **Attention composite** | Time | −0.028 (0.031) | 0.366 | - |
|  | Group × Time | −0.045 (0.044) | 0.321 | 0.151 |
| **Executive Function composite** | Time | −0.007 (0.041) | 0.863 | - |
|  | Group × Time | −0.020 (0.056) | 0.721 | 0.064 |
| Group comparisons were made with the latter group serving as the reference. β represents the standardized regression coefficients. All *p* values are reported before correction for multiple testing.  Abbreviations: Aβ, amyloid-β; AD, Alzheimer’s disease; Ch4p, posterior subdivision of nucleus basalis of Meynert; Ch1/Ch2, medial septal nucleus/vertical limb of the diagonal band of Broca; CU, cognitively unimpaired; SE, standard error. | | | | |

| **eTable 5.** Partial correlation analysis of rates of volume loss in brain regions (Ch4p, Ch1/Ch2, and hippocampus) and rates of change in memory, attention, and executive function composite scores across stable CU Aβ− and emergent AD groups. | | | | |
| --- | --- | --- | --- | --- |
|  | **Stable CU Aβ−** | | **Emergent AD** | |
|  | **Estimate (SE)** | ***p*** | **Estimate (SE)** | ***p*** |
| **Ch4p** |  |  |  |  |
| ***vs Memory*** | 0.076 (0.031) | **0.016** | 0.187 (0.073) | **0.013** |
| ***vs Attention*** | 0.017 (0.011) | 0.124 | 0.057 (0.022) | **0.014** |
| ***vs Executive Function*** | 0.015 (0.009) | 0.110 | 0.035 (0.020) | 0.084 |
| **Ch1/Ch2** |  |  |  |  |
| ***vs Memory*** | 0.042 (0.045) | 0.356 | −0.086 (0.110) | 0.438 |
| ***vs Attention*** | 0.014 (0.016) | 0.395 | −0.030 (0.034) | 0.381 |
| ***vs Executive Function*** | 0.017 (0.013) | 0.190 | 0.021 (0.029) | 0.466 |
| **Hippocampus** |  |  |  |  |
| ***vs Memory*** | 0.098 (0.031) | **0.002** | 0.163 (0.079) | 0.043 |
| ***vs Attention*** | 0.032 (0.011) | **0.004** | 0.019 (0.025) | 0.458 |
| ***vs Executive Function*** | 0.040 (0.009) | **< 0.001** | 0.039 (0.021) | 0.063 |
| Age, sex, and education were included as covariates. All *p* values are reported before correction for multiple testing and are highlighted in bold if they remain statistically significant after correcting for multiple testing.  Abbreviations: Aβ, amyloid-β; AD, Alzheimer’s disease; Ch4p, posterior subdivision of nucleus basalis of Meynert; Ch1/Ch2, medial septal nucleus/vertical limb of the diagonal band of Broca; CU, cognitively unimpaired; SE, standard error. | | | | |

| **eTable 6.** Linear mixed-effects models examining rates of change between stable CU Aβ− *APOE* ε4 carriers and non-carriers. | | | | |
| --- | --- | --- | --- | --- |
|  | **Fixed Effect** | **β (SE)** | ***P-value*** | **Cohen’s *d*** |
| **Stable CU Aβ− Only (n = 328)**  *Group Contrast: ε4 carriers (n = 53) v.s. non-carriers (n = 275)* | | | | |
| **Aβ burden** | Group | 0.039 (0.138) | 0.775 | 0.016 |
|  | Time | −0.057 (0.031) | 0.065 | **-** |
|  | Group × Time | 0.243 (0.079) | **0.002** | **−0.227** |
| **Ch4p volume** | Group | −0.138 (0.151) | 0.361 | 0.051 |
|  | Time | −0.285 (0.027) | **< 0.001** | - |
|  | Group × Time | −0.022 (0.070) | 0.752 | 0.022 |
| **Ch1/Ch2 volume** | Group | −0.097 (0.139) | 0.484 | 0.038 |
|  | Time | −0.140 (0.023) | **< 0.001** | - |
|  | Group × Time | −0.013 (0.058) | 0.817 | 0.016 |
| **Hippocampal volume** | Group | −0.071 (0.145) | 0.625 | 0.027 |
|  | Time | −0.367 (0.022) | **< 0.001** | - |
|  | Group × Time | −0.056 (0.056) | 0.318 | 0.064 |
| **Memory composite** | Group | −0.116 (0.086) | 0.179 | 0.074 |
|  | Time | 0.100 (0.016) | **< 0.001** | - |
|  | Group × Time | 0.008 (0.043) | 0.853 | −0.012 |
| **Attention composite** | Group | −0.281 (0.097) | **0.004** | 0.159 |
|  | Time | −0.067 (0.012) | **< 0.001** | - |
|  | Group × Time | 0.039 (0.034) | 0.250 | −0.086 |
| **Executive Function composite** | Group | −0.142 (0.101) | 0.163 | 0.076 |
|  | Time | −0.015 (0.017) | 0.370 | - |
|  | Group × Time | −0.008 (0.046) | 0.855 | 0.014 |
| Group comparisons were made with the latter group serving as the reference. β represents the standardized regression coefficients. All *p* values are reported before correction for multiple testing. Statistical outputs are highlighted in bold if they remain statistically significant after correcting for multiple testing.  Abbreviations: Aβ, amyloid-β; AD, Alzheimer’s disease; *APOE*, Apolipoprotein E; Ch4p, posterior subdivision of nucleus basalis of Meynert; Ch1/Ch2, medial septal nucleus/vertical limb of the diagonal band of Broca; CU, cognitively unimpaired; SE, standard error. | | | | |

| 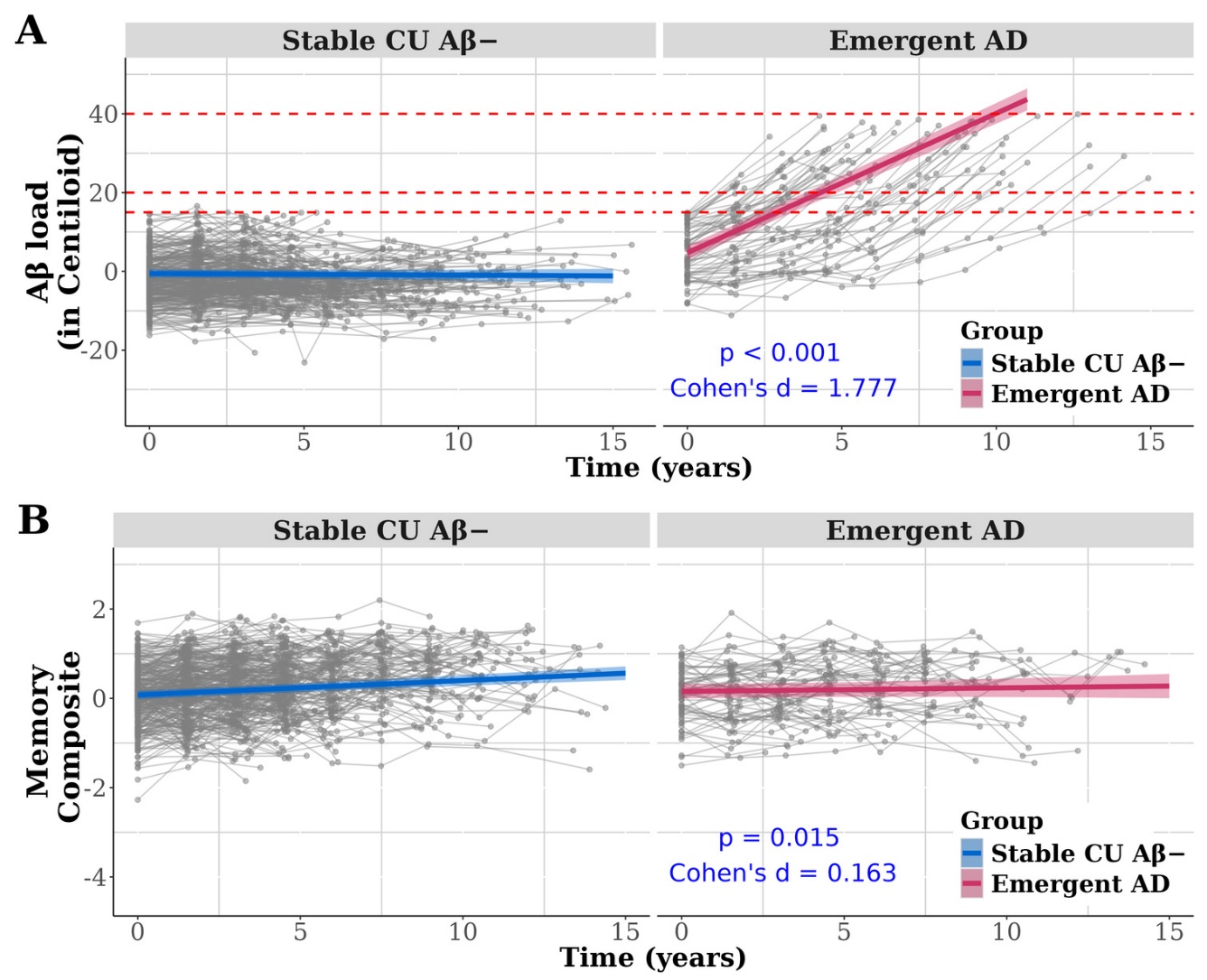 |
| --- |
| **eFigure 1.** Sensitivity analysis assessing the average group trajectories of (A) brain Aβ burden and (B) memory composite scores in the stable CU Aβ− and emergent AD groups, with data restricted to assessments associated with visits where Aβ levels were below 40 CL. The red dashed lines in (A) indicate the levels of Aβ burden at 15, 20, and 40 CL. The grey lines show the individual trajectories of measures for each participant. The coloured lines show the average group trajectories of measures as estimated using the linear mixed-effects model. Abbreviations: Aβ, amyloid-β; Aβ−, Aβ levels < 15 CL; AD, Alzheimer’s disease; CL, Centiloid; CU, cognitively unimpaired. |

| 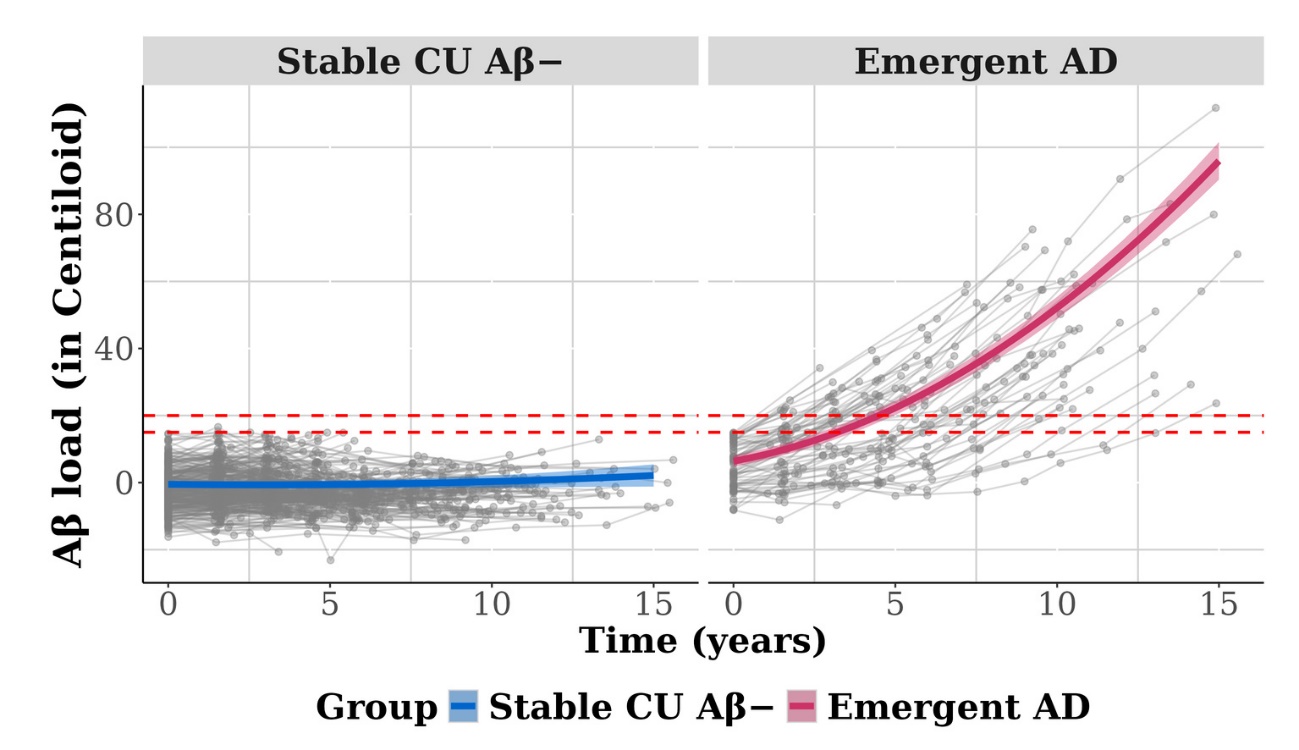 |
| --- |
| **eFigure 2.** Non-linear modelling to longitudinal trajectories of brain Aβ burden for all study participants stratified by group. The red dashed lines indicate the thresholds of Aβ levels at 15 and 20 Centiloids. The grey lines show the individual trajectories of Aβ burden for each participant. The coloured lines show the average group trajectories of Aβ burden as estimated using the non-linear mixed-effects model that accounts for group differences in intercept, slope, and quadratic curvature. Abbreviations: Aβ, amyloid-β; AD, Alzheimer’s disease; CU, cognitively unimpaired. |

| 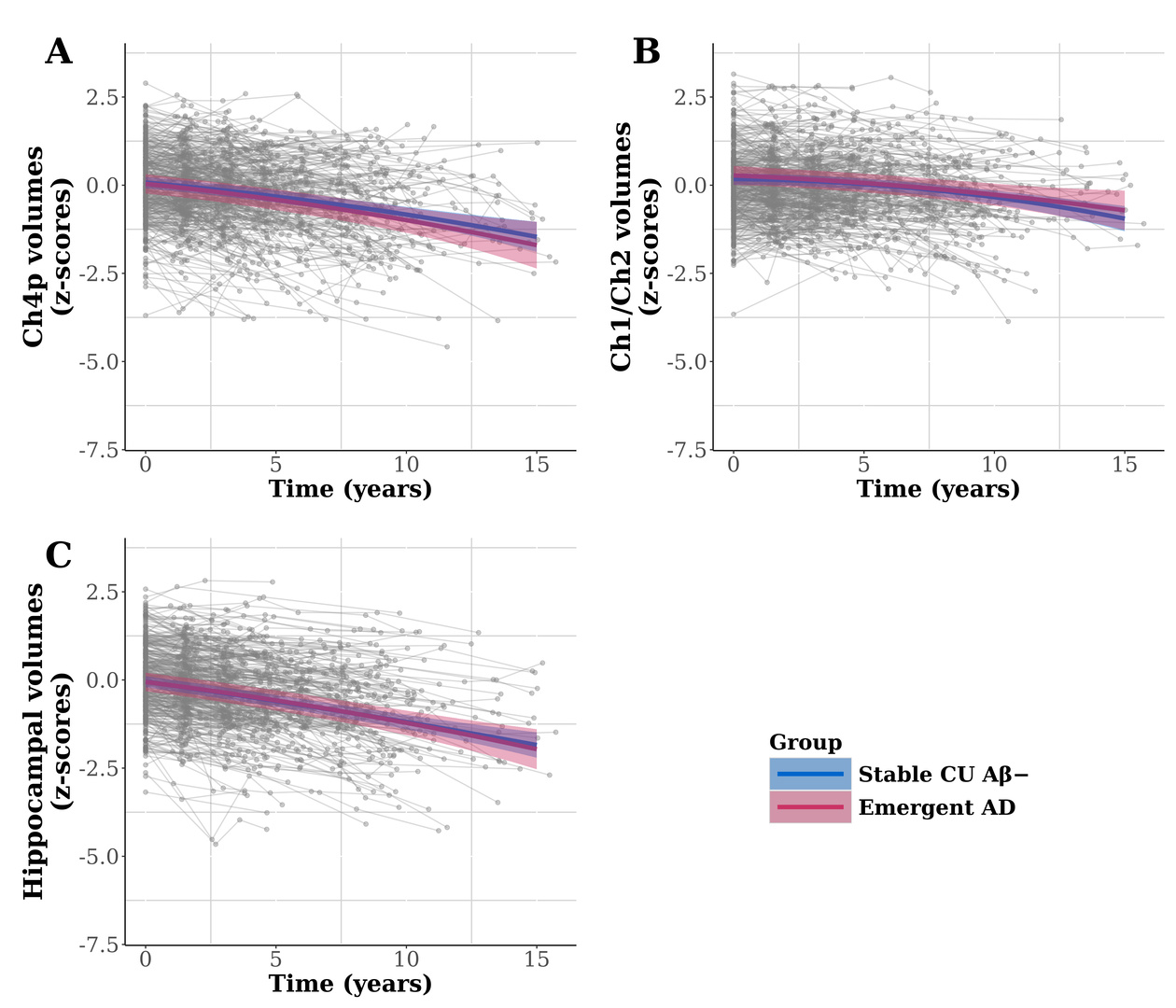 |
| --- |
| **eFigure 3.** Non-linear modelling of longitudinal trajectories for (A) Ch4p, (B) Ch1/Ch2, and (C) hippocampal volumes, stratified by group. The grey lines show the individual trajectories of brain volume for each participant. The coloured lines show the average group trajectories of brain volume as estimated using the non-linear mixed-effects model that accounts for group differences in intercept, slope, and quadratic curvature. Abbreviations: Aβ, amyloid-β; AD, Alzheimer’s disease; Ch4p, posterior subdivision of nucleus basalis of Meynert; Ch1/Ch2, medial septal nucleus/vertical limb of the diagonal band of Broca; CU, cognitively unimpaired. |

| 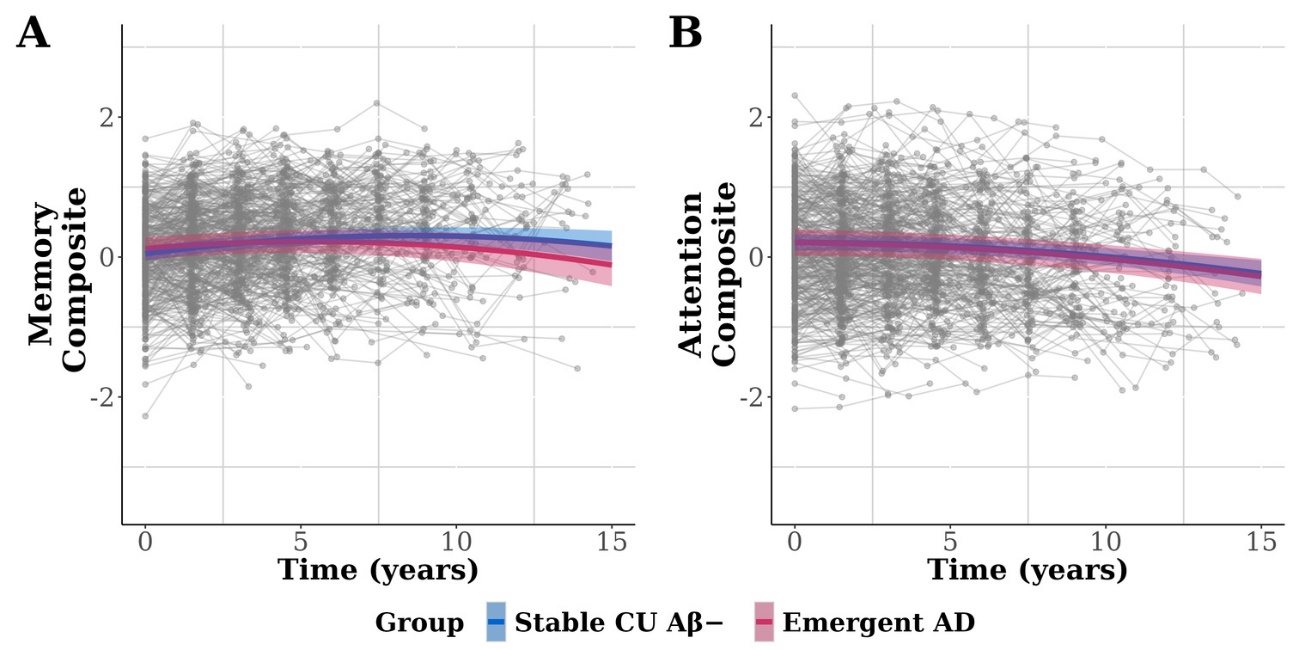 |
| --- |
| **eFigure 4.** Non-linear modelling of longitudinal trajectories for (A) Memory and (B) Attention composite scores, stratified by group. The executive function composite was not included, as its longitudinal data were best fit by a linear model. The grey lines show the individual trajectories of cognitive composite score for each participant. The coloured lines show the average group trajectories of cognitive composite score as estimated using the non-linear mixed-effects model that accounts for group differences in intercept and slope. Abbreviations: Aβ, amyloid-β; AD, Alzheimer’s disease; CU, cognitively unimpaired. |

| 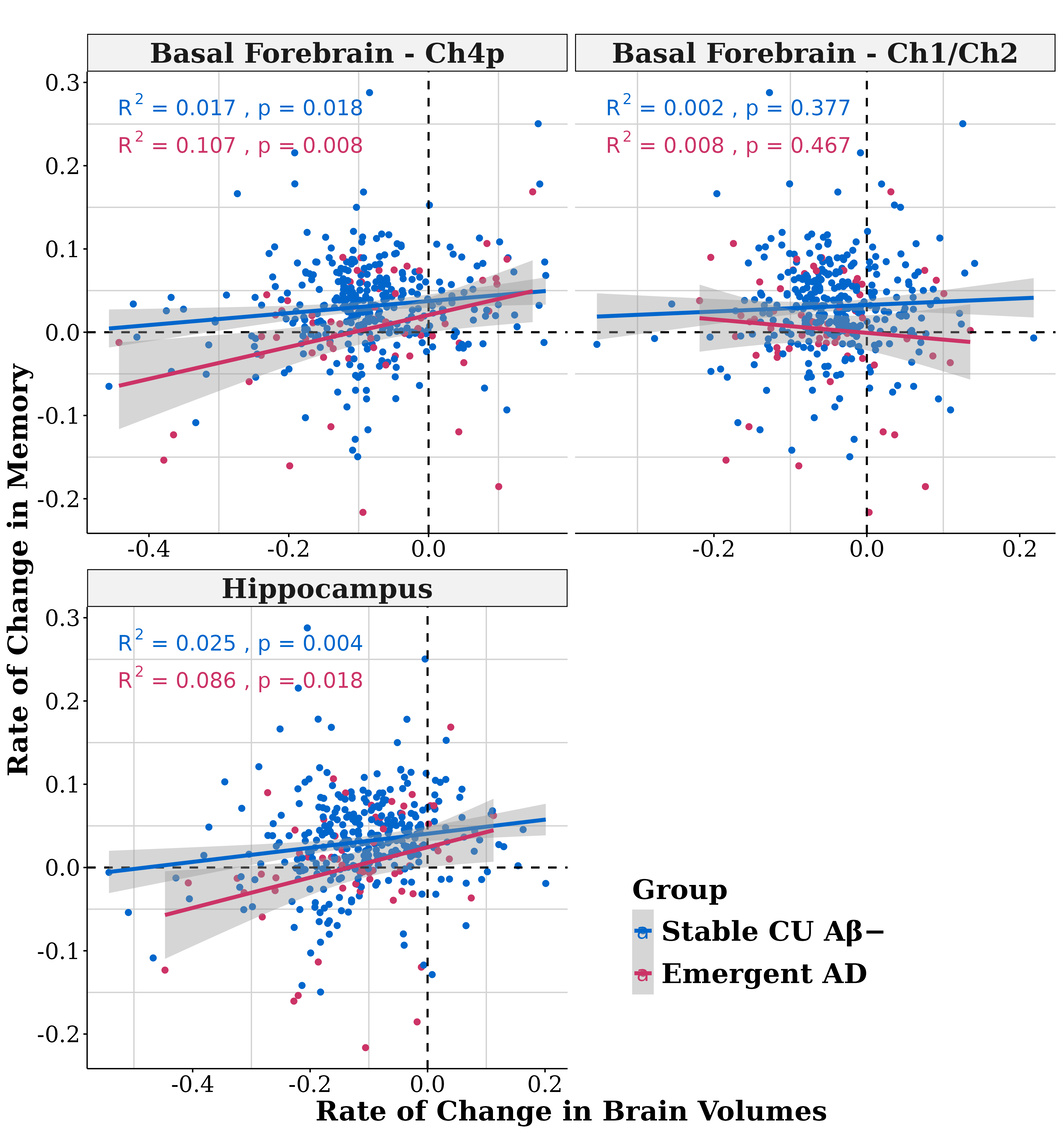 |
| --- |
| **eFigure 5.** Scatterplots showing the associations between the rate of volumetric change in different brain regions and the rate of change in memory, with data points representing stable CU Aβ− individuals (blue) and those with emergent AD (red). Dashed lines indicate reference axes: the horizontal line represents zero change in memory composite scores, and the vertical line represents zero change in brain volumes. Abbreviations: Aβ, amyloid-β; AD, Alzheimer’s disease; Ch4p, posterior subdivision of nucleus basalis of Meynert; Ch1/Ch2, medial septal nucleus/vertical limb of the diagonal band of Broca; CU, cognitively unimpaired. |

| 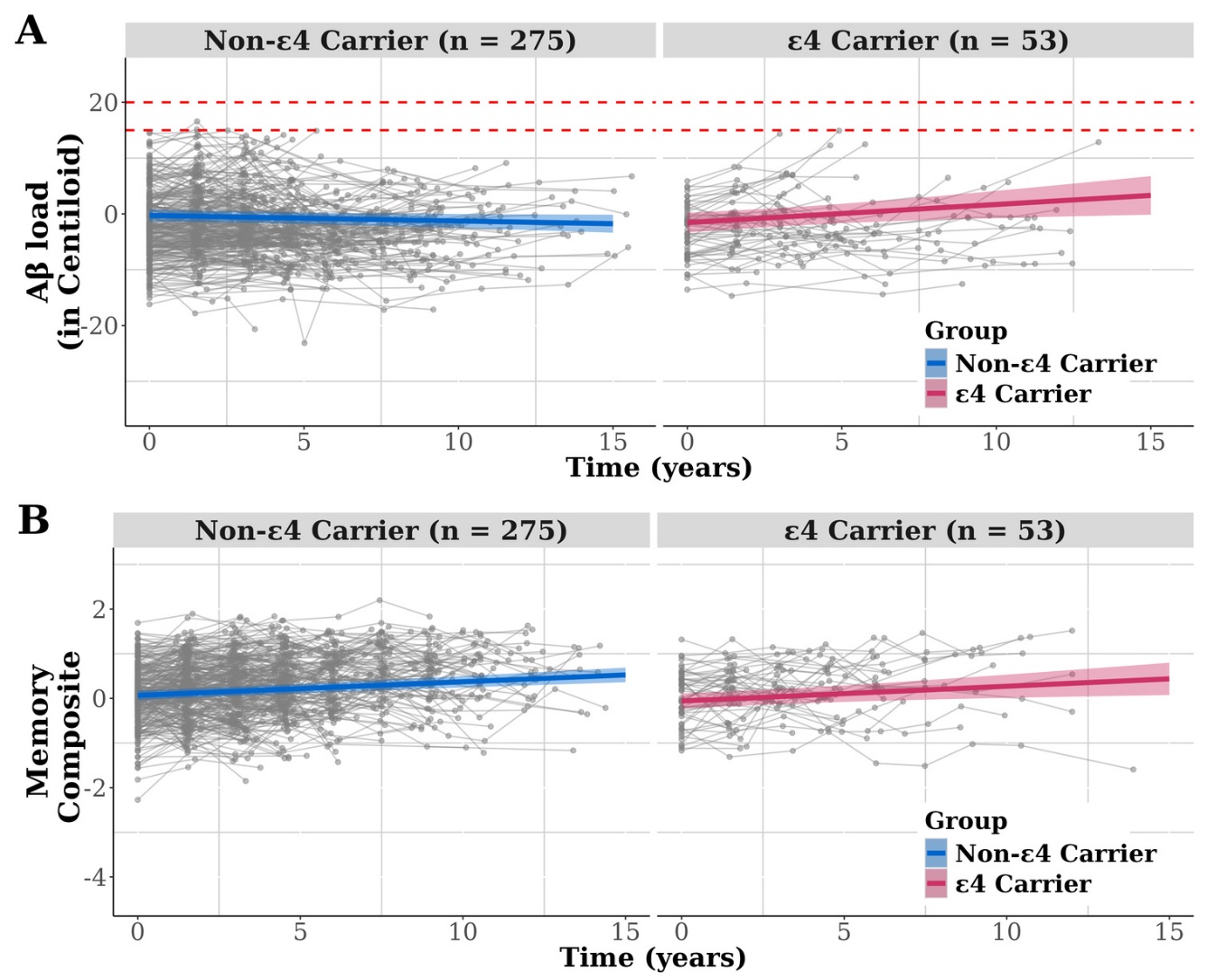 |
| --- |
| **eFigure 6.** Trajectories of (A) brain Aβ burden and (B) memory composite scores for stable CU Aβ− individuals, stratified by *APOE* ε4 carriage. The red dashed lines in (A) indicate the levels of Aβ burden at 15 and 20 CL. The grey lines show the individual trajectories of measures for each participant. The coloured lines show the average group trajectories of measures as estimated using the linear mixed-effects model. Abbreviations: Aβ, amyloid-β; Aβ−, Aβ levels < 15 CL; *APOE*, apolipoprotein E; CL, Centiloid; CU, cognitively unimpaired. |
